# *Streptococcus salivarius* Ss-08 Extracellular Vesicles Suppress OSCC Progression via Inhibition of JAG1–NOTCH1 Signaling

**DOI:** 10.3390/ijms27177595

**Published:** 2026-08-25

**Authors:** Guoding Cao, Meng Yuan, Mingyang Ding, Yichen Jiang, Chongyao Xue, Yong Fang

**Affiliations:** 1 Department of Microbiology, Harbin Medical University, Harbin 150081, China; 2 Heilongjiang Province Key Laboratory of Immunity & Infection, Harbin 150081, China

**Keywords:** extracellular vesicles, oral squamous cell carcinoma, streptococcus salivarius, NOTCH signaling pathway, tumor suppression

## Abstract

*Streptococcus salivarius*-derived extracellular vesicles (SsEVs) have emerged as important mediators of host–microbe communication, but their role in oral squamous cell carcinoma (OSCC) remains unclear. In this study, SsEVs were isolated and characterized by transmission electron microscopy and nanoparticle tracking analysis, and their uptake by CAL-27 cells was confirmed by fluorescence imaging. Functional assays demonstrated that SsEVs inhibited the proliferation, migration, and invasion of CAL-27 cells in a concentration-dependent manner. RNA sequencing revealed substantial transcriptional reprogramming following SsEV treatment, with enrichment analyses indicating the suppression of pathways associated with cell adhesion, extracellular matrix remodeling, lipid metabolism, and particularly Notch signaling. Gene set enrichment analysis (GSEA) and gene set nariant analysis (GSVA) consistently identified Notch signaling as significantly downregulated. Further validation showed that SsEVs markedly decreased the expression of JAG1, NOTCH1, and HEYL at both the mRNA and protein levels. In a CAL-27 xenograft model, SsEV treatment significantly inhibited tumor growth and reduced JAGGED1, NOTCH1, and HEYL expression in tumor tissues, as confirmed by immunohistochemistry. Collectively, these findings demonstrate that SsEVs suppress OSCC progression both in vitro and in vivo by inhibiting the JAG1–NOTCH1–HEYL signaling axis, suggesting that microbiota-derived extracellular vesicles may represent a promising therapeutic approach for OSCC.

## 1. Introduction

OSCC is a highly heterogeneous epithelial malignancy that accounts for approximately 90% of oral cancers. Its initiation and progression have long been attributed to classical risk factors, including tobacco use, alcohol consumption, and human papillomavirus (HPV) infection [1,2]. Despite advances in diagnostic and therapeutic strategies, the clinical outcomes of patients with advanced OSCC remain unsatisfactory due to the high incidence of recurrence and metastasis. Recent studies based on metagenomic sequencing and 16S rRNA profiling have increasingly revealed that OSCC is closely associated with the local microbial ecosystem. Rather than being regarded as a simple accompanying phenomenon, the interaction between the oral microbiota and OSCC is now considered an important regulatory process that influences tumor initiation, progression, and therapeutic resistance through the inflammation, immunity, and metabolism axis [3,4,5,6]. As one of the most microbiologically diverse ecological niches in the human body, the oral cavity harbors a complex microbial community. Increasing evidence indicates that OSCC is accompanied by significant remodeling of the local microbial landscape, characterized by the enrichment of periodontal-associated anaerobic bacteria and the depletion of certain health-associated commensal microorganisms. For example, comparative analyses of paired tumor and adjacent non-tumor tissues have demonstrated increased abundance of *Fusobacterium*, *Prevotella*, and *Parvimonas*, together with decreased levels of commensal genera such as *Streptococcus* and *Rothia* in OSCC tissues, suggesting that oral microbial dysbiosis may contribute to OSCC progression [7,8]. Furthermore, accumulating evidence suggests that certain oral bacteria function not merely as biomarkers of disease, but as active regulators of tumor behavior. *Fusobacterium nucleatum* promotes tumor development through FadA-mediated activation of the E-cadherin/β-catenin signaling pathway and Fap2-mediated recognition of tumor-associated glycans [9,10]. Recent studies have further demonstrated that *F. nucleatum* enriched in invasive regions of OSCC facilitates glucose transporter type 1 (GLUT1) membrane localization through the GalNAc–autophagy–TBC1D5 axis, thereby enhancing glycolysis and lactate production and inducing tumor-associated macrophage (TAM) formation to promote OSCC progression [11]. In addition, *Porphyromonas gingivalis* promotes OSCC cell stemness acquisition and tumor formation by upregulating SCD1-mediated lipid synthesis via the NOD1/KLF5 axis [12]. Collectively, these findings demonstrate that oral microorganisms can regulate tumor development through specific molecular mechanisms.

However, current studies investigating the tumor-promoting or tumor-suppressive effects of microorganisms have mainly focused on direct interactions between intact bacteria and host cells, whereas the mechanisms by which bacteria transmit regulatory signals through non-contact pathways remain poorly understood. In recent years, bacterial extracellular vesicles (BEVs) have emerged as important mediators of microbe–host communication. BEVs can be produced by both Gram-negative and Gram-positive bacteria and contain diverse bioactive components, including proteins, lipids, nucleic acids, and metabolites. These vesicles can be internalized by host cells and subsequently regulate inflammatory responses, immune functions, and cellular signaling states [13,14]. Compared with live bacteria, BEVs can achieve long-distance molecular delivery without requiring bacterial colonization, suggesting that they may represent a novel regulatory mode through which microorganisms influence the tumor microenvironment. Although the roles of pathogen-derived extracellular vesicles in infectious and inflammatory diseases have been extensively investigated, whether extracellular vesicles derived from oral commensal bacteria can regulate tumor progression remains largely unknown.

*Streptococcus salivarius* is an important commensal bacterium involved in maintaining oral microbial homeostasis. Among its strains, *S. salivarius* K12 has attracted considerable attention due to its ability to produce bacteriocins and inhibit potential oral pathogens [15,16]. A recent randomized double-blind clinical trial further demonstrated that *S. salivarius* K12 reduced the incidence and severity of radiotherapy-associated oral mucositis in patients with head and neck malignancies. This effect was associated with the inhibition of opportunistic pathogens and restoration of commensal microbial communities, suggesting an important ecological regulatory role of *S. salivarius* K12 in tumor-associated oral environments [17]. Recent studies have further confirmed that *S. salivarius* can produce extracellular vesicles, indicating that these vesicles may mediate more complex regulatory effects on host cells [18]. However, whether extracellular vesicles derived from *Streptococcus salivarius* can directly influence OSCC progression and the underlying molecular mechanisms remain unclear. Therefore, this study systematically investigated the effects of SsEVs on the biological behaviors of OSCC and further elucidated the molecular mechanisms underlying their tumor-suppressive effects. This study provides new insights into the role of oral commensal bacteria-derived nanostructures in tumor regulation.

## 2. Result

### 2.1. Isolation, Purification, and Internalization of SsEVs by OSCC Cells

**Figure 1:**
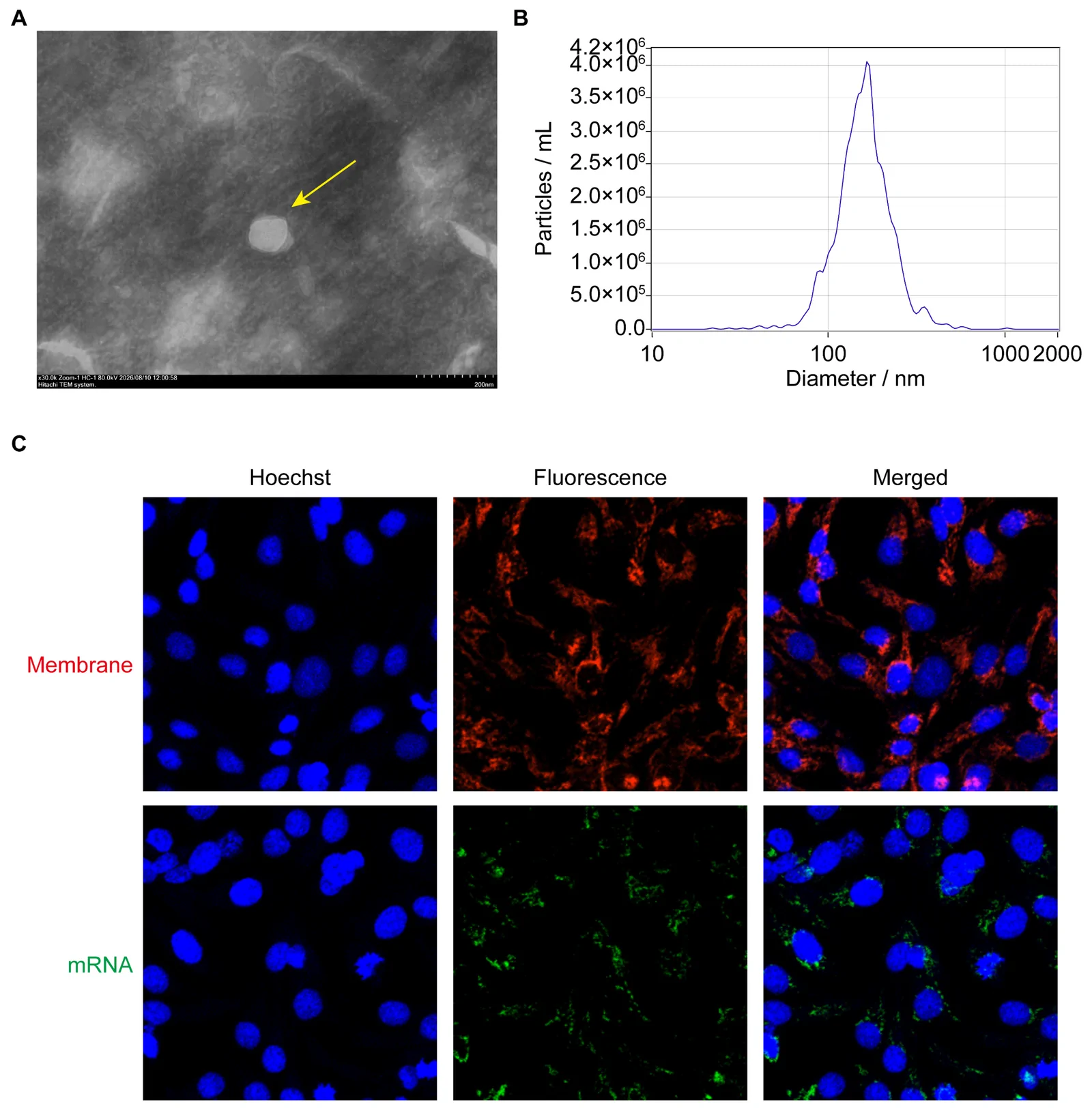
Identification and uptake analysis of SsEVs. (**A**) TEM image showing the typical morphology of purified SsEVs. Scale bar, 200 nm. *n* = 3. (**B**) NTA profile showing the size distribution of SsEVs from a single replicate experiment. The reported median size and concentration values were calculated from this specific measurement. *n* = 3. (**C**) Representative confocal microscopy images showing the cellular uptake of SsEVs by CAL-27 cells. *n* = 3. The membrane and RNA components of SsEVs were labeled with the ExoGlow™-Membrane EV Labeling Kit and the ExoGlow™-RNA EV Labeling Kit, respectively. Scale bar, 10 µm.

To investigate the role of SsEVs in OSCC, Streptococcus salivarius was first isolated and identified from oral microbiota samples. Bacterial cultures in the logarithmic growth phase were collected, and bacterial cells and debris were removed by sequential centrifugation. SsEVs were then enriched and purified by ultracentrifugation. Transmission electron microscopy showed that the purified SsEVs exhibited round or oval membrane bound vesicular structures with heterogeneous sizes (Figure 1A, Figure S1). Nanoparticle tracking analysis revealed median particle diameters of 156.1 nm, 189.9 nm, and 191.9 nm across three independent replicates, which were derived from the representative measurements (Figure 1B, Figure S2). To assess whether SsEVs could be taken up by OSCC cells, the membrane and RNA components of SsEVs were fluorescently labeled using the ExoGlow™-Membrane EV Labeling Kit and ExoGlow™-RNA EV Labeling Kit (System Biosciences, USA), respectively, and then incubated with CAL-27 cells. To rule out the interference of probe self-aggregation and dye micelle formation, appropriate control experiments were performed by incubating cells with PBS alone (without probes or EVs) and with dye-only (without EVs). Both control groups were observed under wide-field fluorescence microscopy, revealing no aggregated fluorescent signals or false-positive background in either condition, which is consistent with the well-established stability of these probes in previous studies (Figure S3). Confocal microscopy showed that labeled SsEVs were internalized by CAL-27 cells and were mainly distributed in the cytoplasmic and perinuclear regions (Figure 1C). These results indicate that purified SsEVs display typical extracellular vesicle morphology and size characteristics and can be internalized by OSCC cells.

### 2.2. SsEVs Suppress Proliferation, Migration and Invasion of CAL-27 Cells

**Figure 2:**
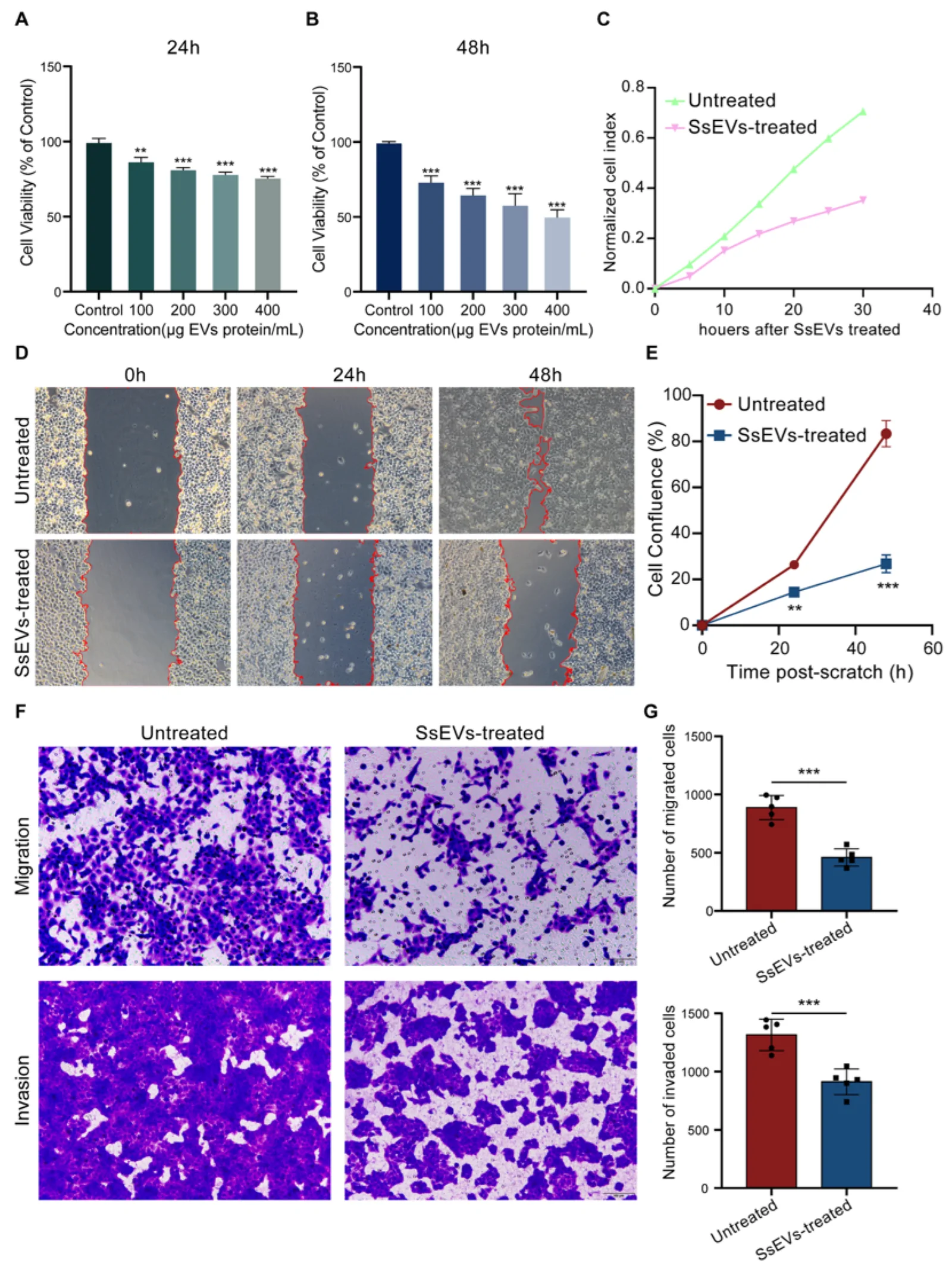
SsEVs suppress CAL-27 cell viability, migration, and invasion. (**A**) CCK-8 assay showing the viability of CAL-27 cells after 24 h of treatment with different concentrations of SsEVs (µg EVs protein/mL). *n* = 3. (**B**) CCK-8 assay showing the viability of CAL-27 cells after 48 h of treatment with different concentrations of SsEVs (µg EVs protein/mL). *n* = 3. (**C**) Real-time cell analysis using the iCELLigence system showing reduced growth of CAL-27 cells following SsEV treatment. *n* = 3. (**D**) Representative images of wound healing assays in the control and SsEV-treated CAL-27 cells. (**E**) Quantitative analysis of wound closure. *n* = 3. (**F**) Representative images of Transwell migration and invasion assays in the control and SsEV-treated CAL-27 cells. Scale bar, 100 µm. (**G**) Quantification of migrated and invaded cells. Data are presented as mean ± SD. *n* = 5. ** *p* < 0.01, *** *p* < 0.001.

To evaluate the effects of SsEVs on malignant phenotypes of OSCC cells, CAL-27 cells were incubated with different concentrations of SsEVs, and cell viability was examined at different time points. CCK-8 assays showed that treatment with varying concentrations of SsEVs (ranging from 0, 100, 200, 300, 400 µg EVs protein/mL) reduced CAL-27 cell viability in a concentration-dependent manner compared with the cell group (Figure 2A). The inhibitory effect of SsEVs was more pronounced after 48 h of treatment than after 24 h of treatment (Figure 2B). Among the tested concentrations, 400 μg/mL SsEVs showed the strongest inhibitory effect on cell viability and was therefore used for subsequent functional assays. Real-time cell analysis using the iCelligence system further showed that 400 μg/mL SsEVs markedly slowed the increase in the CAL-27 cell growth curve, as reflected by reduced cell index progression (Figure 2C). We next assessed the effects of SsEVs on CAL-27 cell migration and invasion. Wound healing assays showed that wound closure was markedly reduced in the SsEV group at both 24 h and 48 h compared with the cell group, indicating impaired migratory capacity (Figure 2D,E). Transwell assays further showed that SsEV treatment significantly decreased the number of migrated and invaded CAL-27 cells (Figure 2F,G). These results indicate that SsEVs suppress the proliferative, migratory, and invasive capacities of CAL-27 cells.

### 2.3. RNA Sequencing Reveals Distinct Transcriptional Reprogramming of OSCC Cells Following SsEV Treatment

**Figure 3:**
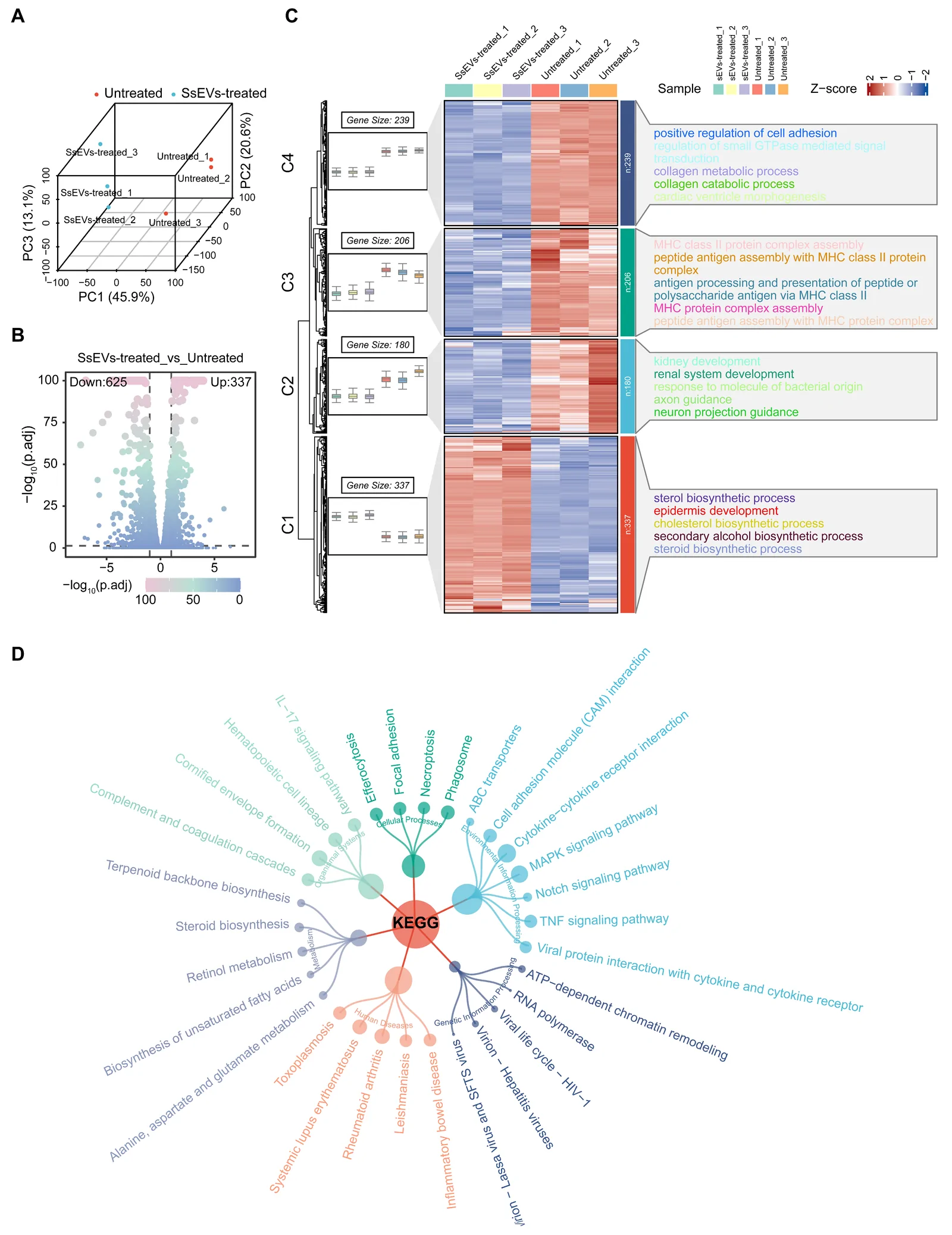
RNA sequencing reveals SsEVs induced transcriptional remodeling in CAL-27 cells. (**A**) PCA of RNA sequencing profiles from the untreated and SsEV treated CAL-27 cells. (**B**) Volcano plot showing differentially expressed genes between the untreated and SsEV treated CAL-27 cells. (**C**) Expression trend clustering and GO biological process enrichment analysis of differentially expressed genes. DEGs were divided into four major expression modules, and representative enriched biological processes are shown for each module. (**D**) KEGG pathway enrichment analysis of all differentially expressed genes, showing cancer related and signal transduction pathways affected by SsEV treatment.

To characterize the molecular changes induced by SsEV treatment at the transcriptomic level, RNA sequencing was performed in treated SsEVs and untreated CAL-27 cells. PCA showed a clear separation between the SsEV treated group and the untreated group, indicating that SsEV treatment induced marked transcriptomic changes in CAL-27 cells (Figure 3A). Differential expression analysis identified 962 differentially expressed genes, including 337 upregulated and 625 downregulated genes (Figure 3B). Expression trend clustering further classified the DEGs into four major modules, reflecting distinct transcriptional responses to SsEV treatment. Functional annotation showed that the downregulated modules were mainly enriched in biological processes related to the regulation of cell adhesion, small GTPase mediated signal transduction, collagen metabolism, axon guidance, and sterol/cholesterol biosynthesis, suggesting that SsEVs may affect transcriptional programs associated with cell motility, extracellular matrix remodeling, and lipid biosynthesis. In contrast, the upregulated module was enriched in response to molecule of bacterial origin, MHC class II complex assembly, and antigen processing and presentation, suggesting that SsEVs induced bacterial stimulus associated and immune related transcriptional changes (Figure 3C). KEGG enrichment analysis of all DEGs further showed that multiple cancer related and signal transduction pathways were affected, including focal adhesion, cell adhesion molecules, MAPK signaling pathway, TNF signaling pathway, and Notch signaling pathway (Figure 3D). These results indicate that SsEV treatment reshapes transcriptional programs related to cell adhesion, matrix remodeling, lipid metabolism, and bacterial stimulus response in OSCC cells, providing a basis for the further investigation of Notch signaling.

### 2.4. SsEVs Suppress the JAG1–NOTCH1–HEYL Signaling Axis in CAL-27 Cells

**Figure 4:**
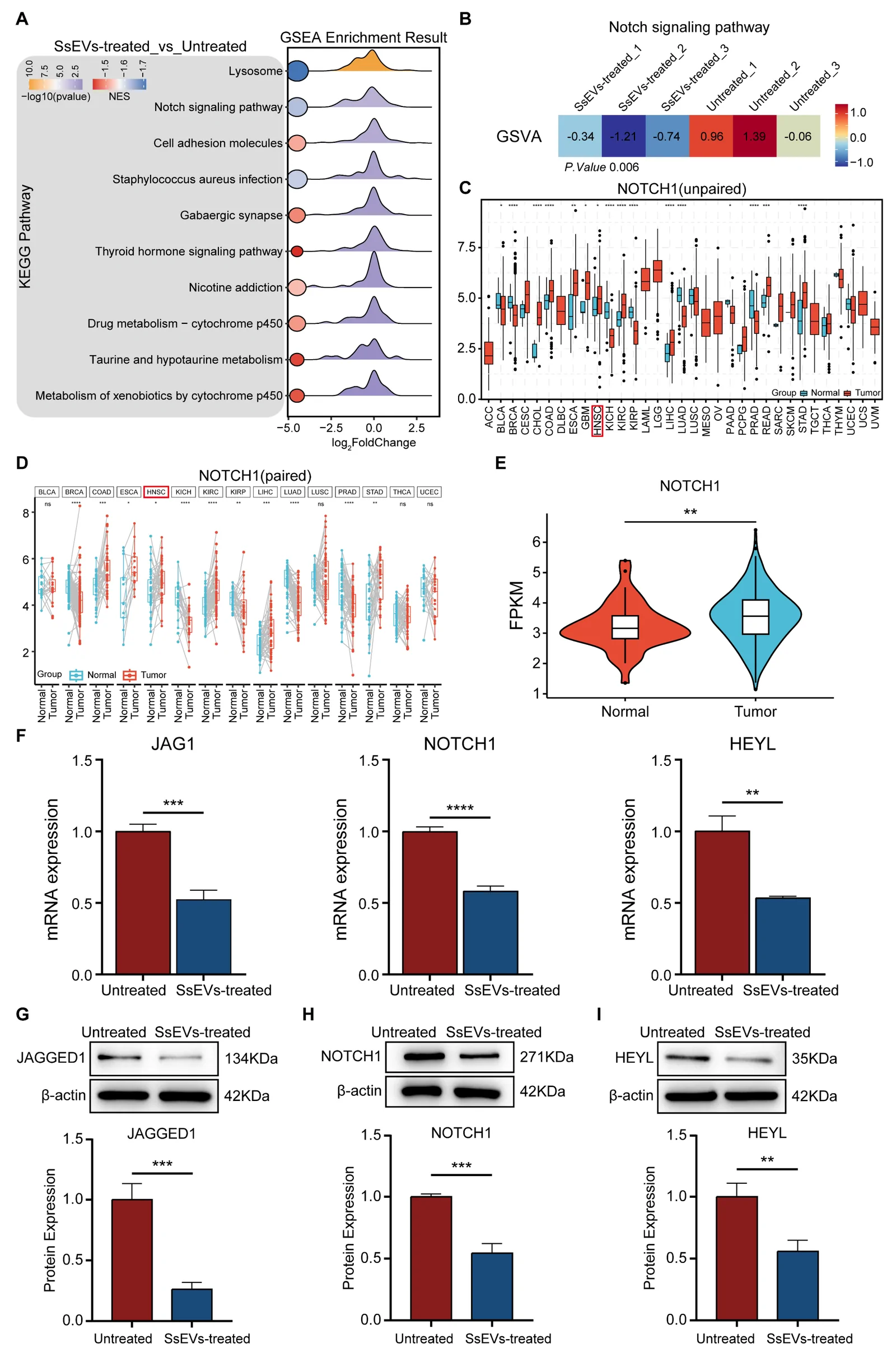
SsEV treatment suppresses NOTCH1 signaling activity and JAG1–NOTCH1–HEYL axis expression in OSCC cells. (**A**) GSEA ridge plot showing the top downregulated KEGG pathways in SsEV treated CAL-27 cells compared with untreated cells. (**B**) GSVA score of the Notch signaling pathway in the untreated and SsEV treated CAL-27 cells. (**C**) Pan cancer analysis of NOTCH1 expression in tumor and normal tissues based on unpaired samples. (**D**) Paired analysis of NOTCH1 expression in tumor and matched normal tissues across cancer types, with HNSC highlighted. (**E**) NOTCH1 expression in normal (*n* = 44) and OSCC tumor tissues (*n* = 341). (**F**) RT-qPCR analysis of JAG1, NOTCH1, and HEYL mRNA expression in untreated and SsEV treated CAL-27 cells. *n* = 3. (**G**–**I**) Western blot analysis of JAGGED1, NOTCH1, and HEYL protein expression in untreated and SsEV treated CAL-27 cells. β-Actin was used as the loading control. Quantification of JAGGED1, NOTCH1, and HEYL protein expression was based on Western blot analysis. Data are presented as mean ± SD. “ns” indicates no significant difference, * *p* < 0.05, ** *p* < 0.01, *** *p* < 0.001, **** *p* < 0.0001.

Given that KEGG enrichment analysis identified multiple cancer-related and signal transduction pathways among the DEGs, including the Notch signaling pathway, we next performed GSEA to determine the direction of pathway-level changes after SsEV treatment. GSEA revealed that the Notch signaling pathway was ranked among the top downregulated pathways in SsEV-treated cells, suggesting that Notch signaling represents a suppressed candidate pathway following SsEV exposure (Figure 4A). Consistently, GSVA analysis showed a significantly lower Notch signaling pathway score in the SsEV-treated group than in the untreated group, indicating reduced Notch pathway activity at the sample level (Figure 4B). To evaluate the disease relevance of NOTCH1 in OSCC, we next analyzed NOTCH1 expression using public databases. Pan-cancer analysis showed dysregulated NOTCH1 expression across multiple tumor types. In the HNSC cohort, both unpaired and paired analyses revealed differential NOTCH1 expression between tumor and normal tissues (Figure 4C,D). Further analysis focused on OSCC samples showed significantly higher NOTCH1 expression in tumor tissues than in normal tissues, suggesting a potential association between NOTCH1 and OSCC progression (Figure 4E). We then examined the expression of key Notch pathway components in SsEV-treated CAL-27 cells. RT-qPCR analysis showed that SsEV treatment significantly decreased the mRNA levels of JAG1, NOTCH1, and HEYL (Figure 4F). Western blot analysis further confirmed that the JAGGED1 (encoded by JAG1), NOTCH1, and HEYL protein levels were markedly reduced after SsEV treatment, and densitometric quantification was consistent with the changes observed in the immunoblot bands (Figure 4G–I). Together, these findings indicate that SsEV treatment reduces Notch pathway activity and suppresses the JAG1–NOTCH1–HEYL signaling axis at both the transcriptional and protein levels, providing a molecular basis for the reduced proliferation, migration, and invasion of OSCC cells following SsEV treatment.

### 2.5. SsEVs Inhibit OSCC Xenograft Growth and Attenuate JAG1–NOTCH1–HEYL Axis In Vivo

**Figure 5:**
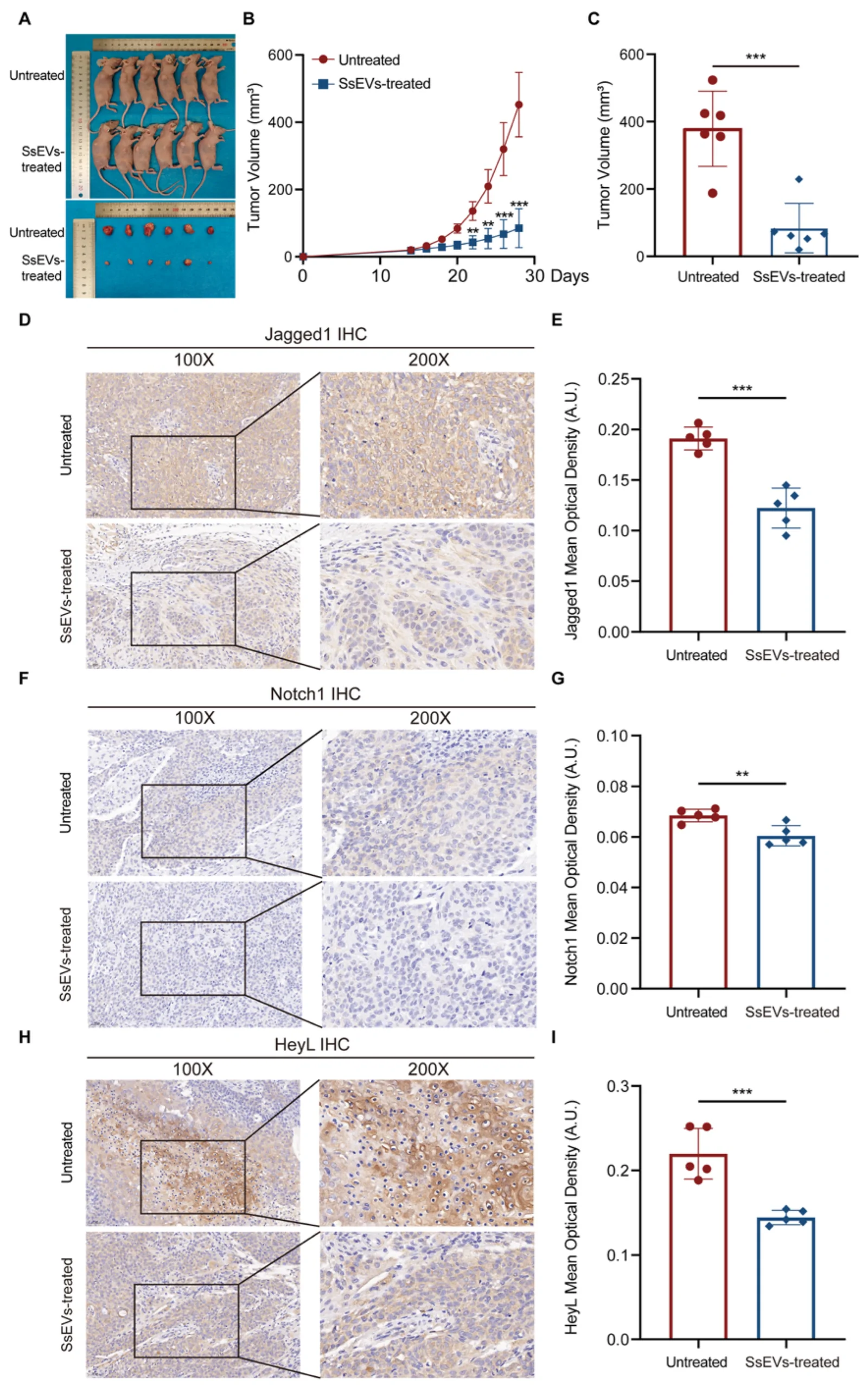
SsEVs inhibit OSCC xenograft growth and suppress JAGGED1–NOTCH1–HEYL axis expression in vivo. (**A**) Representative images of nude mice bearing CAL-27 xenograft tumors and excised tumors from the untreated and SsEV treated groups. (**B**) Tumor growth curves showing tumor volume changes over time in the untreated and SsEV treated groups. *n* = 5. (**C**) Quantification of tumor volume at the endpoint. *n* = 5. (**D**) Representative IHC staining images of JAGGED1 in xenograft tumor tissues. Images are shown at 100× and 200× magnification. (**E**) Quantification of JAGGED1 staining based on mean optical density. *n* = 5. (**F**) Representative IHC staining images of NOTCH1 in the xenograft tumor tissues. Images are shown at 100× and 200× magnification. (**G**) Quantification of NOTCH1 staining based on mean optical density. *n* = 5. (**H**) Representative IHC staining images of HEYL in xenograft tumor tissues. Images are shown at 100× and 200× magnification. (**I**) Quantification of HEYL staining based on mean optical density. *n* = 5. Data are presented as mean ± SD. ** *p* < 0.01, *** *p* < 0.001.

To further determine whether SsEVs exert antitumor effects in vivo, a CAL-27 xenograft model was established in nude mice. Compared with the untreated group, SsEV treatment markedly reduced tumor growth, as shown by the smaller tumor size and slower increase in tumor volume over time (Figure 5A,B). At the endpoint, tumors from the SsEV treated group showed significantly lower tumor volume than those from the untreated group (Figure 5C), indicating that SsEVs suppress OSCC tumor growth in vivo. We next examined whether the NOTCH1 signaling axis was also affected in xenograft tumors. IHC staining showed weaker JAGGED1, NOTCH1, and HEYL signals in tumors from the SsEV treated group compared with the control group (Figure 5D,F,H). Quantitative analysis further confirmed significant reductions in the mean optical density of JAGGED1, NOTCH1, and HEYL staining after SsEV treatment (Figure 5E,G,I). These in vivo results demonstrate that SsEVs inhibit OSCC xenograft growth accompanied by suppression of the JAG1–NOTCH1–HEYL axis, supporting its involvement in the antitumor effect of SsEVs.

## 3. Discussion

In the present study, we identified extracellular vesicles derived from Streptococcus salivarius as negative regulators of OSCC progression. SsEVs exhibited typical nanoscale vesicular morphology and were efficiently internalized by CAL-27 cells. Functionally, SsEVs inhibited the proliferation, migration, and invasion of OSCC cells in vitro and suppressed the growth of xenograft tumors in vivo. Mechanistically, SsEVs attenuated NOTCH signaling activity, as evidenced by the reduced expression of key components of the NOTCH pathway and its downstream effectors, suggesting that suppression of this pathway may contribute to the antitumor effects of SsEVs. These findings expand the current understanding of oral bacterial vesicles beyond their conventional role in pathogenicity and suggest that extracellular vesicles derived from commensal bacteria can actively modulate the behavior of malignant epithelial cells. This concept is particularly important because accumulating evidence has demonstrated that bacterial extracellular vesicles are not passive cellular debris but biologically active nanoparticles carrying proteins, nucleic acids, lipids, and microbe-associated molecular patterns. Following internalization by host cells, these vesicles can regulate signaling programs involved in inflammation, immunity, metabolism, and carcinogenesis [19].

The major conceptual advance of this study is the demonstration of the functional heterogeneity of oral bacterial extracellular vesicles. In our previous study, we showed that extracellular vesicles derived from Streptococcus mutans (SmEVs) promoted OSCC progression by activating the β-catenin/TCF7/Fra-1 signaling axis [20]. In contrast, the present study demonstrates that SsEVs exert the opposite biological effects, accompanied by suppression of the NOTCH1–JAG1–HEYL signaling axis. Together, these findings indicate that the biological functions of oral bacterial extracellular vesicles are species dependent, with vesicles derived from different bacterial species exerting distinct, and even opposing, effects on tumor cells. Such functional divergence is not an isolated phenomenon. Among the best-characterized periodontal pathogens in OSCC, *Porphyromonas gingivalis*-derived outer membrane vesicles (OMVs) have been shown to promote OSCC proliferation, migration, and invasion by suppressing ferroptosis through activation of the NF-κB signaling pathway, thereby inducing epithelial–mesenchymal transition (EMT) [21]. In addition, sRNA23392 packaged within *P. gingivalis* OMVs further enhances tumor cell migration and invasion by directly downregulating the expression of desmocollin-2 (DSC2) [22]. Other mechanisms have also been proposed. For example, *P. gingivalis* OMVs may contribute to OSCC development through the coordinated downregulation of multiple genes, including TNFSF15, ZNF292, ATRX, ASPM, and KIF20B [23]. Notably, even extracellular vesicles derived from the same bacterial species may exert divergent biological effects. For example, Metsäniitty et al. reported that extracellular vesicles derived from *Fusobacterium nucleatum* inhibited the proliferation of metastatic OSCC cells and induced apoptosis while simultaneously enhancing their migratory capacity [24]. In contrast, Chen et al. demonstrated that *F. nucleatum* outer membrane vesicles markedly promoted oral cancer metastasis both in vitro and in vivo by activating autophagic flux [25]. This phenomenon, in which extracellular vesicles derived from the same bacterial species produce opposing effects in the same disease model, strongly suggests that the biological activities of bacterial extracellular vesicles are not determined solely by bacterial identity, but rather result from the combined effects of intrinsic bacterial characteristics, vesicular cargo composition, and the physiological state of recipient cells. More importantly, extracellular vesicles derived from a limited number of oral bacterial species have even exhibited potential tumor-suppressive properties. In the same study, Metsäniitty et al. further demonstrated that extracellular vesicles derived from *Aggregatibacter actinomycetemcomitans* reduced the proliferation, invasion, and vasculogenic mimicry of metastatic OSCC cells while promoting apoptosis, primarily through the actions of cytolethal distending toxin and lipopolysaccharide O antigen [24]. Furthermore, extracellular vesicles derived from the recently identified OSCC-enriched bacterium *Stomatobaculum* longum have been reported to promote OSCC malignant progression through activation of the BRCA1/EXO1/TP53BP1 signaling axis [26]. Collectively, these findings suggest that the biological effects of bacterial extracellular vesicles represent the net output of multiple signaling components integrated by recipient cells rather than a simple reflection of bacterial species identity. Accordingly, microbial dysbiosis associated with OSCC should not be interpreted merely as an increase in tumor-promoting bacteria, but rather as a functional imbalance resulting from the simultaneous enhancement of protumorigenic signals and attenuation of tumor-suppressive signals.

Mechanistically, transcriptomic analysis revealed that SsEV treatment induced extensive transcriptional reprogramming in CAL-27 cells. Downregulated genes were predominantly enriched in pathways associated with cell adhesion, focal adhesion, collagen metabolism, small GTPase-mediated signaling, and lipid and cholesterol biosynthesis, whereas upregulated genes were mainly enriched in pathways involved in the response to bacterial stimuli and antigen processing. Notably, among genes associated with the NOTCH signaling pathway, the expression of the ligand JAG1, the receptor NOTCH1, and the downstream effector HEYL was significantly reduced following SsEV treatment. The oncogenic role of JAG1 in OSCC has been well-documented. JAG1 is frequently overexpressed in both OSCC cell lines and clinical specimens, and its elevated expression is closely associated with poor patient prognosis [27]. Mechanistically, as a key ligand of the NOTCH1 receptor, JAG1 drives cancer stem cell properties and pulmonary metastasis in OSCC through the FAS–ERK–JAG1–NOTCH1 signaling axis. Activation of FAS promotes ERK phosphorylation, which in turn upregulates JAG1 expression and activates NOTCH signaling, whereas silencing of either JAG1 or NOTCH1 suppresses tumorsphere formation in vitro and tumor growth in vivo [28]. In addition, JAG1 serves as a central convergence point for multiple non-coding RNA regulatory networks. For example, circ_0068162 relieves miR-186-mediated repression of JAG1 through a sponge mechanism, whereas lncRNA *XIST* promotes tumor migration and proliferation through the miR-124/JAG1 competing endogenous RNA regulatory axis. Although initiated by distinct upstream regulatory pathways, these mechanisms ultimately converge on JAG1 to promote OSCC progression [29,30]. The role of NOTCH signaling in cancer is complex and highly context dependent. Depending on tumor type, cellular state, and the surrounding microenvironment, NOTCH signaling may function as either an oncogenic driver or a tumor suppressor pathway [31,32]. In OSCC and head and neck squamous cell carcinoma (HNSCC), dysregulation of the NOTCH pathway has been associated with cancer stemness, epithelial plasticity, invasion, and therapeutic response. However, pathway inhibition may also induce compensatory signaling dependencies, such as activation of the JAK-STAT pathway [28,33,34,35,36]. Our findings further showed that SsEV treatment reduced not only the expression of the ligand JAG1, but also that of the receptor NOTCH1. This observation has important functional implications. Compared with reducing ligand availability alone, the simultaneous downregulation of both the ligand and its receptor may produce a more profound suppression of NOTCH signaling through two complementary mechanisms. On the one hand, decreased JAG1 expression limits ligand-mediated activation of NOTCH1. On the other hand, reduced NOTCH1 expression restricts receptor-mediated signal transduction even in the presence of residual ligand. The combined effects of these two mechanisms are ultimately reflected by the marked downregulation of the downstream effector HEYL. As a direct transcriptional target of the NOTCH intracellular domain (NICD), HEYL expression directly reflects the activation status of the NOTCH signaling pathway [37,38,39]. Notably, HEYL has been reported to exert oncogenic functions in multiple tumor types. In breast cancer, HEYL overexpression promotes angiogenesis and tumor growth by upregulating proangiogenic factors including CXCL1, CXCL2, and CXCL3, whereas the inhibition of HEYL markedly suppresses tumor growth [40]. In contrast, HEYL has been identified as a candidate tumor suppressor in hepatocellular carcinoma, where it promotes p53-mediated apoptosis through the upregulation of P53 expression, highlighting the tissue-specific functions of HEYL [41]. In OSCC, NOTCH pathway components, including HEY1, a member of the same transcription factor family as HEYL, are consistently upregulated in both cell lines and clinical specimens, and pharmacological inhibition of NOTCH signaling suppresses OSCC growth in vitro [42]. Collectively, these findings suggest that HEYL and its related family members are likely to exert protumorigenic functions in OSCC and that their downregulation may contribute to suppression of the malignant phenotype. Therefore, the coordinated downregulation of JAG1, NOTCH1, and HEYL following SsEV treatment provides multiple lines of evidence supporting the notion that SsEVs exert their antitumor effects, at least in part, through inhibition of the JAG1–NOTCH signaling pathway.

This study also has potential translational implications. Extracellular vesicles derived from commensal bacteria may provide a cell-free strategy for microbiota-based cancer intervention. In recent years, bacterial extracellular vesicles have attracted increasing attention as naturally occurring nanomaterials for disease treatment, and their translational potential in cancer immunotherapy and drug delivery has been widely recognized [43]. In particular, tumor vaccines based on bacterial outer membrane vesicles have entered clinical evaluation, whereas extracellular vesicles derived from probiotic bacteria have also been proposed as a promising therapeutic platform capable of conferring health benefits while avoiding the risks associated with bacterial colonization and infection [44,45]. Compared with live bacterial administration, purified extracellular vesicles offer theoretical advantages in terms of controllability, safety, and compatibility with nanoscale drug delivery systems [46]. A recent randomized double-blind clinical trial demonstrated that administration of live *Streptococcus salivarius* K12 reduced the incidence of severe radiotherapy-induced oral mucositis in patients with head and neck cancer, supporting the clinical feasibility of *S. salivarius*-based interventions in the supportive management of cancer therapy, although that study did not investigate extracellular vesicles [17]. Our findings provide mechanistic evidence supporting the potential value of extracellular vesicles as functional effectors of commensal bacteria and offer proof-of-concept for the future development of cell-free antitumor strategies based on commensal bacteria-derived extracellular vesicles.

Several limitations should be considered. First, the co-injection model used in this study primarily captures the effect of SsEVs on early tumor establishment; it does not fully replicate the therapeutic challenge of treating pre-existing tumors. Evaluating SsEV administration in established tumor models therefore remains an important direction for future work. Second, the fetal bovine serum employed here was processed by triple 100 nm filtration rather than ultracentrifugation-based EV depletion. Although the consistent functional outcomes across independent experiments suggest that any residual bovine EVs did not appreciably alter the results, adopting EV-depleted serum would further strengthen the rigor of subsequent studies. Third, universally accepted protein markers for assessing the identity and purity of Gram-positive bacterial EVs are still lacking. Unlike eukaryotic EVs, for which tetraspanins serve as canonical positive markers, bacterial vesicles naturally carry a mixture of membrane-associated and cytoplasmic proteins, complicating the straightforward application of conventional purity criteria. Despite these limitations, the convergence of TEM, NTA, and functional data provides reliable evidence for the nature and anti-tumor activity of the SsEVs characterized in this work.

## 4. Materials and Methods

### 4.1. Culture of Streptococcus salivarius and Isolation of SsEVs

The strain of Streptococcus salivarius Ss-08 used in this study was previously isolated from oral microbiota samples and identified by the Department of Microbiology, Harbin Medical University and has been deposited in the China Center for Type Culture Collection (CCTCC) under CCTCC No. M20261361. A bacterial suspension containing 1.5 × 10^4^ CFU/mL of Streptococcus salivarius was inoculated into eight sterile conical flasks, each containing 45 mL of brain heart infusion (BHI) medium, and cultured statically at 37 °C for 16 to 18 h. After the initial culture, the bacterial cells were harvested by centrifugation at 5000 rpm (corresponding to approximately 3000 to 4000× *g* according to the specifications of the fixed-angle rotor used in our centrifuge) at 4 °C for 20 min, and the supernatant was discarded while the pellet was resuspended. The resuspended cells were subsequently transferred into eight culture flasks containing 400 mL of fresh BHI medium and further incubated statically at 37 °C for 16 to 18 h to obtain sufficient bacterial products. The resulting bacterial culture was distributed into 50 mL centrifuge tubes and centrifuged at 5000 rpm at 4 °C for 20 min to remove the bacterial cells, and the supernatant was retained. The supernatant was centrifuged once more under identical conditions (5000 rpm, 4 °C, 20 min) to further eliminate residual bacterial debris and large particles. Following centrifugation, the supernatant was aspirated and filtered through a 0.45 μm PVDF microporous membrane. A small aliquot of the filtrate was plated on a BHI agar plate and incubated at 37 °C to confirm the absence of viable bacterial growth (Figure S4). The sterile filtrate was then distributed into ultracentrifuge tubes (with a maximum volume of 38 mL per tube) and subjected to ultracentrifugation at 120,000× *g* for 60 min at 4 °C. After ultracentrifugation, the supernatant was discarded and the resulting pellets were collected. The pellets from multiple ultracentrifugation runs were pooled into a single tube, gently resuspended and washed with 2 mL of sterile PBS, and the volume was adjusted to 38 mL with sterile PBS. The sample was then ultracentrifuged again at 120,000× *g* and 4 °C for 60 min for further purification. The final supernatant was discarded, and the resulting pellet, defined as *Streptococcus salivarius*-derived extracellular vesicles (SsEVs), was resuspended in 200 μL of sterile PBS, aliquoted, and stored at −80 °C until further use.

### 4.2. Transmission Electron Microscopy (TEM)

Purified SsEVs were resuspended in PBS at a final concentration of 800 μg/mL. An aliquot of the SsEV suspension was placed onto a 300-mesh carbon coated copper grid, allowed to adsorb, and negatively stained. After air drying, the morphology of SsEVs was observed using a transmission electron microscope (Hitachi High-Technologies Corporation, Tokyo, Japan).

### 4.3. Nanoparticle Tracking Analysis (NTA)

The particle size distribution and concentration of SsEVs were measured by the Zeta View nanoparticle tracking analysis system (Particle Metrix GmbH, Inning am Ammersee, Germany). SsEV samples were diluted in sterile PBS to an appropriate concentration range and loaded into the NTA system. Measurements were performed at 25 °C, and the hydrodynamic diameter and particle concentration of the SsEVs were calculated based on Brownian motion tracking.

### 4.4. RNA Sequencing and Bioinformatic Analysis

CAL-27 cells were seeded in 6-well plates and treated after cell attachment. Cells in the experimental group were incubated with culture medium containing 400 μg/mL SsEVs, whereas control cells received an equal volume of fresh DMEM. After incubation for 48 h at 37 °C in a humidified atmosphere with 5% CO_2_, cells were washed two to three times with PBS and lysed with 600 μL TRIzol reagent per well. Total RNA samples were snap-frozen in liquid nitrogen, stored at −80 °C, and then sent to Majorbio Bio-Pharm Technology Co., Ltd. (Shanghai, China) for RNA extraction, quality assessment, library construction, and high-throughput sequencing. Total RNA was extracted using TRIzol reagent, and RNA quality and integrity were assessed using an Agilent 2100 Bioanalyzer (Agilent Technologies, Santa Clara, CA, USA). Raw sequencing data were processed by standard quality control, sequence alignment, and expression quantification procedures. Differentially expressed genes (DEGs) were identified using the thresholds of |fold change| > 2 and adjusted *p* value < 0.05. Expression trend clustering, Gene Ontology (GO) functional annotation, and Kyoto Encyclopedia of Genes and Genomes (KEGG) pathway enrichment analysis were performed based on the identified DEGs. Gene Set Enrichment Analysis (GSEA) was further applied to the whole-gene expression matrix to evaluate pathway-level changes. Data analysis and visualization were performed using the online platform www.bioinformatics.com.cn (accessed on 9 July 2026). RNA-Seq data for OSCC tissues and paired normal tissues were obtained from The Cancer Genome Atlas (TCGA) database and used to compare NOTCH1 expression between tumor and normal tissues.

### 4.5. Cell Culture

The human OSCC cell line CAL-27 was maintained at the Department of Microbiology, Harbin Medical University (Harbin, China). CAL-27 cells were cultured in high-glucose Dulbecco’s modified Eagle’s medium (DMEM) supplemented with 10% fetal bovine serum (CB FBS, catalog no. AUS-01E-02; collected from healthy cattle in non-epidemic areas, sterile-collected, pooled, and processed through triple 100 nm filtration; certified free of mycoplasma and bovine viruses including BVDV, PI3, IBR, and BPV), 100 U/mL penicillin, and 0.1 mg/mL streptomycin. Cells were maintained at 37 °C in a humidified incubator with 5% CO_2_. Of note, the FBS used in this study was not subjected to ultracentrifugation-based depletion of extracellular vesicles; accordingly, the potential presence of trace bovine EVs cannot be excluded.

### 4.6. SsEV Labeling and Cellular Uptake Assay

CAL-27 cells were seeded on coverslips in 24-well plates at a density of 5 × 10^5^ cells/mL and cultured overnight to allow cell attachment. For membrane labeling, purified SsEVs were labeled using the ExoGlow^TM^-Membrane EV Labeling Kit (System Biosciences, LLC, Palo Alto, CA, USA) according to the manufacturer’s instructions. Briefly, the reaction buffer and membrane labeling dye were mixed and incubated with 100 μg SsEVs for 30 min at room temperature in the dark. For RNA labeling, SsEVs were labeled using the ExoGlow™-RNA EV Labeling Kit (System Biosciences, LLC, Palo Alto, CA, USA). SsEVs were mixed with the reaction buffer and RNA labeling dye, followed by incubation at 37 °C for 60 min protected from light. The labeled SsEVs were then added to fresh culture medium and incubated with CAL-27 cells for 24 h at 37 °C with 5% CO_2_. After incubation, cells were stained with Hoechst to visualize nuclei, washed to remove excess dye, and examined under a Cell Voyager CV1000 confocal imaging system (Yokogawa Electric Corporation, Tokyo, Japan).

### 4.7. Cell Viability and Real-Time Proliferation Assays

Cell viability was assessed using the CCK-8 assay (APExBIO Technology LLC, Houston, TX, USA). CAL-27 cells were seeded in 96-well plates at a density of 3 × 10^4^ cells/well in 100 μL medium and cultured overnight at 37 °C with 5% CO_2_. After cell attachment, cells were treated with complete medium containing 100, 200, 300, or 400 μg/mL SsEVs, while control cells received an equal volume of fresh complete medium. After 48 h of incubation, CCK-8 reagent was added at 10% of the culture volume and incubated for 1 h at 37 °C. Absorbance was measured at 450 nm using a microplate reader. Real-time cell proliferation was monitored using the iCELLigence system. CAL-27 cells were seeded into E-Plate 16 plates at 3 × 10^4^ cells/well with a final volume of 200 μL per well. After stable cell attachment, different concentrations of SsEVs were added to the experimental wells, whereas control wells received an equal volume of PBS. Cells were continuously monitored for 72 h at 37 °C with 5% CO_2_, and cell index values were automatically recorded to evaluate the cell growth dynamics.

### 4.8. Wound Healing Assay

The wound healing assay was performed to evaluate the effect of SsEVs on CAL-27 cell migration. CAL-27 cells were seeded in 6-well plates and cultured until approximately 90% confluence. A linear wound was generated in the cell monolayer using a sterile 200 μL pipette tip. After gently washing with PBS to remove detached cells, control cells were cultured in serum-free DMEM, whereas SsEV-treated cells were cultured in serum-free DMEM containing 400 μg/mL SsEVs. Cells were maintained at 37 °C with 5% CO_2_, and wound areas were photographed at 0 h, 24 h, and 48 h. Changes in wound width were measured using ImageJ 1.53e (National Institutes of Health, Bethesda, MD, USA) to assess cell migration.

### 4.9. Migration and Invasion Assays

Cell migration and invasion were evaluated using Transwell chambers with an 8.0 μm pore size (Corning Incorporated, Corning, NY, USA). For the migration assay, CAL-27 cells suspended in 200 μL serum-free DMEM were seeded into the upper chamber and treated with SsEVs (400 μg/mL), while the lower chamber was filled with DMEM containing 10% fetal bovine serum (FBS) as a chemoattractant. For the invasion assay, the upper chamber inserts were pre-hydrated with serum-free DMEM at 37 °C for 2 h prior to seeding. Cells were then plated in the upper chamber under serum-free conditions with SsEVs (400 μg/mL), and the lower chamber was supplemented with DMEM containing 10% FBS. After incubation at 37 °C for 72 h, non-migrated or non-invaded cells on the upper surface were gently removed with cotton swabs. Cells that migrated or invaded to the lower surface were fixed with 4% paraformaldehyde (Biosharp, Hefei, China) and stained with 0.1% crystal violet (Biosharp, Hefei, China). Stained cells were visualized and imaged using an inverted microscope (Leica Microsystems, Wetzlar, Germany). Quantification was performed by counting cells in five randomly selected fields per insert using ImageJ 1.53e (National Institutes of Health, Bethesda, MD, USA). All experiments were conducted in at least three independent biological replicates.

### 4.10. Quantitative Real-Time PCR (qRT-PCR)

Total RNA was extracted using TRIzol reagent and reverse transcribed into cDNA using Prime Script™ RT Master Mix (Takara Bio Inc., Kusatsu, Shiga, Japan) according to the manufacturer’s instructions. Quantitative real-time PCR (qRT-PCR) was performed using SYBR Premix Ex Taq™ (Takara Bio Inc., Kusatsu, Shiga, Japan). The thermocycling conditions were as follows: initial denaturation at 95 °C for 10 s, followed by 45 cycles of 98 °C for 10 s, 60 °C for 20 s, and 72 °C for 20 s. Relative gene expression was calculated using the 2^−ΔΔCt^ method with GAPDH as the internal control. Primer sequences are listed in Table 1.

### 4.11. Western Blotting

Total cellular protein was extracted using RIPA lysis buffer (Beyotime Biotechnology, Shanghai, China), and protein concentrations were determined by the BCA assay (Beyotime Biotechnology, Shanghai, China). Proteins were separated by SDS-PAGE and transferred to nitrocellulose membranes. Membranes were blocked with 5% nonfat milk at room temperature for 2 h and incubated with primary antibodies (diluted in TBST) overnight at 4 °C. Primary antibodies included anti-β-actin (Proteintech, 20536–1-AP, 1:10,000), Notch1 (Bioss, bsm-60871R, 1:1000), Jagged1 (Bioss, bs-1448R, 1:1000), and HeyL (Bioss, bs-16499R, 1:2000). After washing, membranes were incubated with HRP-conjugated secondary antibodies (A0208, A0216; Beyotime, 1:2000) at room temperature for 1 h. Signals were developed using ECL reagents (Biosharp, Hefei, China), and band intensities were quantified with ImageJ 1.53e (National Institutes of Health, Bethesda, MD, USA).

### 4.12. Animal Experiments

Female BALB/c nude mice aged 4–5 weeks were purchased from Liaoning Changsheng Biotechnology Co., Ltd. (Benxi, Liaoning, China). All mice were housed under specific-pathogen-free conditions with sterilized bedding, food, and water, and were allowed to acclimatize for at least one week before the experiment. All animal procedures were reviewed and approved by the Institutional Animal Ethics Committee of Harbin Medical University (approval no. HMUIRB2025026) and were performed in accordance with institutional animal welfare guidelines and ARRIVE guidelines. The mice were randomly divided into two groups, with six mice in each group. Ear tags were used for individual identification. The control group received CAL-27 cells alone, and the SsEV-treated group received CAL-27 cells mixed with SsEVs. Briefly, CAL-27 cells were prepared at 1 × 10^6^ cells in 0.15 mL PBS. For the SsEV-treated group, CAL-27 cells were mixed with SsEVs at a final concentration of 400 μg/mL, with a total injection volume of 0.15 mL. The prepared cell suspensions were kept on ice, gently mixed before injection, and slowly injected subcutaneously into the posterior region of the right axilla of each mouse. Tumor formation and mouse general condition were monitored regularly. After tumors became palpable, tumor length and width were measured every two days using calipers. Tumor volume was calculated using the following formula: tumor volume = 0.5 × longest diameter × shortest diameter^2^. The experiment was terminated when tumor size approached but did not exceed 15 mm in maximum diameter, according to institutional animal welfare requirements. At the endpoint, mice were deeply anesthetized with 5% isoflurane and euthanized by cervical dislocation. Death was confirmed by the absence of reflexes and cessation of breathing before tissue collection. Tumors were then excised, photographed, weighed, and measured. The collected tumor tissues were fixed, embedded, and sectioned for subsequent histological and immunohistochemical analyses.

### 4.13. Immunohistochemistry (IHC)

Tumor tissues were fixed, paraffin-embedded, and sectioned. After deparaffinization and rehydration, sections were treated with 3% hydrogen peroxide for 30 min at room temperature to block endogenous peroxidase activity. Following antigen retrieval and blocking of non-specific binding, sections were incubated overnight at 4 °C with primary antibodies against NOTCH1 (1:500), JAGGED1 (1:200), and HEYL (1:200). After washing, slides were incubated with HRP-conjugated secondary antibodies (Bio-anti-rabbit IgG) at 37 °C for 30 min. Immunoreactivity was developed using 3,3′-diaminobenzidine (DAB) and counterstained with hematoxylin. Stained sections were examined under a light microscope (Leica Microsystems, Wetzlar, Germany). For each sample, one randomly selected field were captured at 100× magnification. Protein expression levels were quantified by measuring mean optical density using ImageJ 1.53e (National Institutes of Health, Bethesda, MD, USA). All images were acquired and analyzed under identical exposure and threshold settings to ensure comparability across groups.

### 4.14. Statistical Analysis

Data were analyzed and plotted using GraphPad Prism 8.0 (GraphPad Software, San Diego, CA, USA). Results are presented as mean ± standard deviation (mean ± SD). For comparisons between two groups, statistical significance was assessed using an independent-samples *t*-test. For multiple group comparisons, one-way ANOVA followed by Dunnett’s or Tukey’s post hoc test was used. Statistical significance was set at *p* < 0.05.

## 5. Conclusions

In conclusion, this study demonstrates that extracellular vesicles derived from *Streptococcus salivarius* suppress OSCC cell proliferation, migration, and invasion in vitro and inhibit xenograft tumor growth in vivo. Mechanistically, these antitumor effects are associated with the inhibition of NOTCH signaling through the coordinated downregulation of JAG1, NOTCH1, and HEYL. In contrast to the tumor-promoting effects of extracellular vesicles derived from *Streptococcus mutans*, our findings further highlight the functional heterogeneity of oral bacterial extracellular vesicles and redefine OSCC-associated microbial dysbiosis as a bidirectional imbalance characterized by enhanced protumorigenic signaling and diminished protective signaling. These findings suggest that tumor progression is driven not only by the acquisition of pathogenic bacterial signals, but also by the loss of protective vesicle-mediated communication derived from commensal bacteria.

## Figures and Tables

**Table 1:** Primer sequences used for qRT-PCR.

NOTCH1-F	GATGGCCTCAATGGGTACAAG
NOTCH1-R	TCGTTGTTGTTGATGTCACAGT
JAG1-F	ACCAGAACGGCAACAAAACT
JAG1-R	GACCCATGCTTGGGACTG
HEYL-F	CAGCCCTTCGCAGATGCAA
HEYL-R	CCAATCGTCGCAATTCAGAAAG
GAPDH-F	ATCACTGCCACCCAGAAGGAC
GAPDH-R	TTTCTAGACGGCAGGTCAGG

## Data Availability

The original contributions presented in this study are included in the article/Supplementary Material. Further inquiries can be directed to the corresponding author.

## Supplementary Materials

The following supporting information can be downloaded at: https://www.mdpi.com/article/10.3390/ijms27177595/s1.
