# Supplementary material for: Streptococcus salivarius Ss-08 Extracellular Vesicles Suppress OSCC Progression via Inhibition of JAG1–NOTCH1 Signaling
Source: Int J Mol Sci. 2026 Aug 25;27(17):7595. doi: 10.3390/ijms27177595 (PMC13566742; doi:10.3390/ijms27177595)
Supplement: Supplementary file 1 [file ijms-27-07595-s001.zip › ijms-4462129-supplementary.pdf]

Supplementary material

Figure S1. TEM image showing the typical morphology of purified SsEVs.

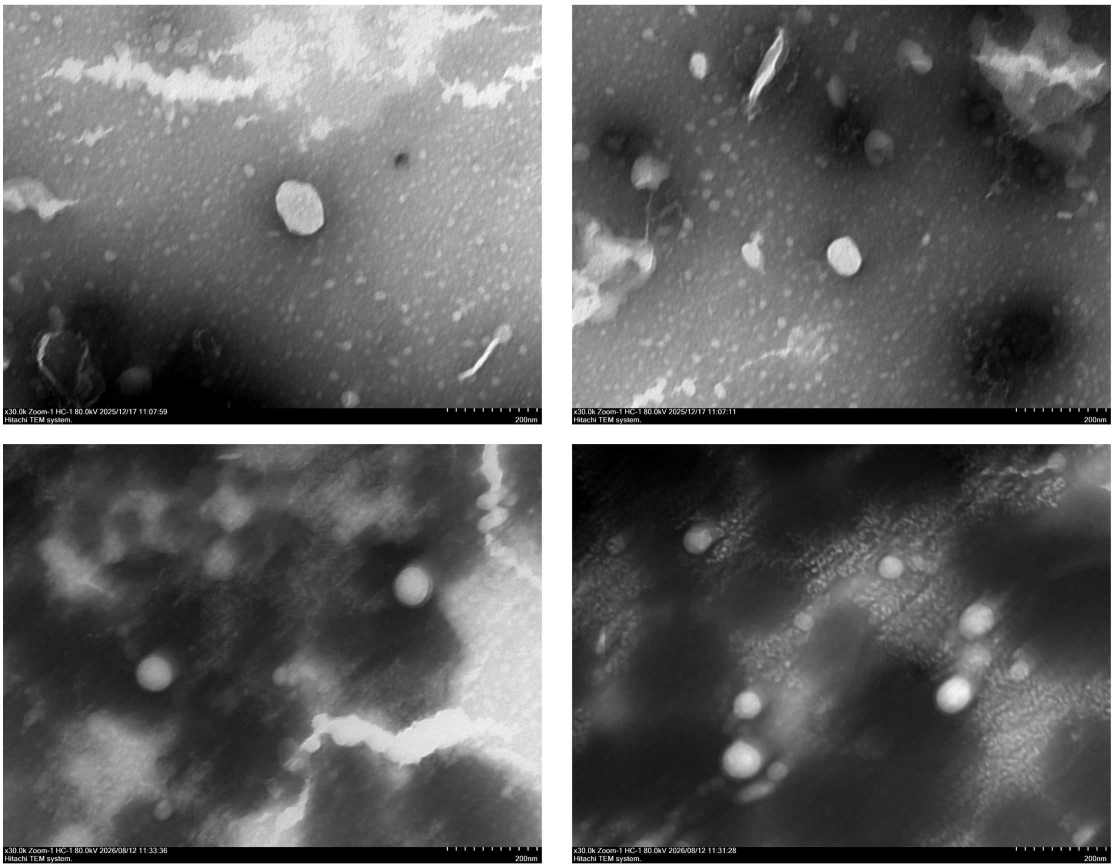

Figure S1: Representative transmission electron microscopy (TEM) images of the isolated EVs demonstrating their typical spherical morphology and heterogeneous size distribution (scale bars = 200 nm).

Figure S2. NTA profile showing the size distribution of SsEVs.

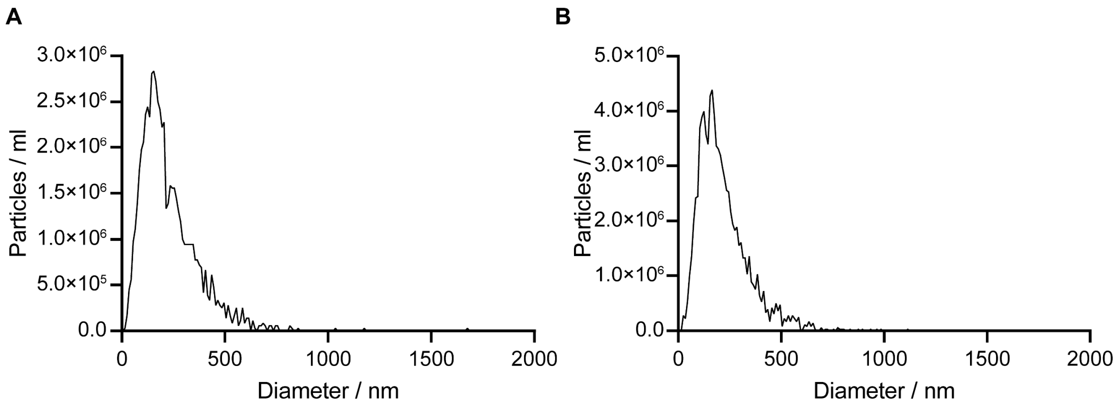

Figure S2 A-B: Representative nanoparticle tracking analysis (NTA) size distribution curves of the isolated EVs, showing two independent replicates from a total of three independent experiments.

Figure S3. Negative control experiments for the cellular uptake assay.

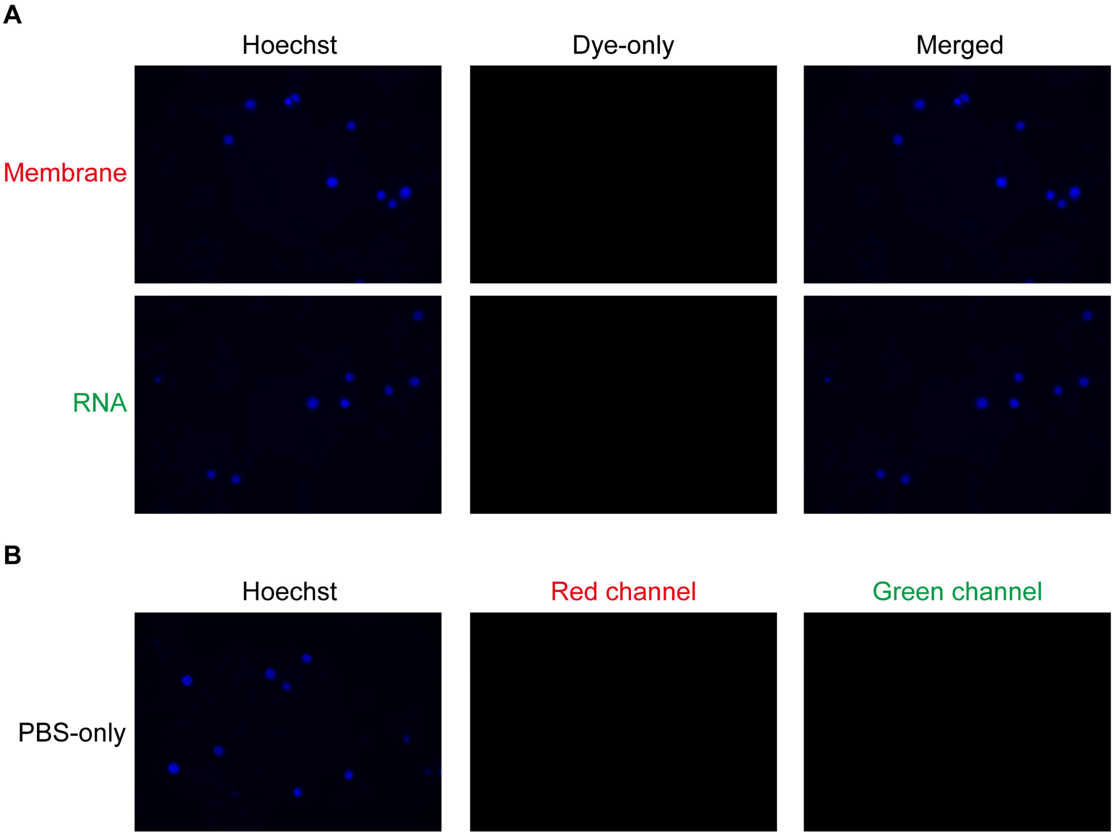

Figure S3. Negative control experiments. (A) Representative images of CAL-27 cells treated with dye-only without EVs to exclude non-specific probe binding and micelle formation. (B) Representative images of CAL-27 cells treated with PBS-only to exclude cellular autofluorescence. Hoechst-stained nuclei are shown in blue, and both panels confirm the absence of detectable signals in the green and red channels. The membrane and RNA components of SsEVs were labeled with the ExoGlow™-Membrane EV Labeling Kit and the ExoGlow™-RNA EV Labeling Kit, respectively. Scale bar, 50µm.

Figure S4. Sterility testing of purified SsEVs

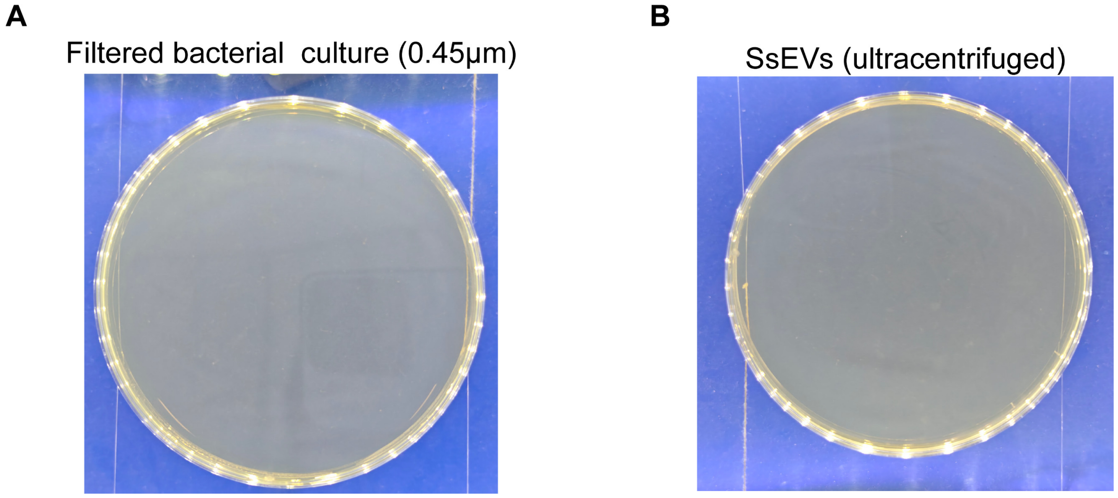

Figure S4. Assessment of live bacterial carryover in SsEVs preparations. (A) Bacterial culture filtered through a 0.45-μm pore-size membrane, demonstrating complete retention of bacteria with no visible colonies. (B) Final SsEVs preparation after three rounds of ultracentrifugation, similarly showing no bacterial growth. These results confirm that the combination of 0.45-μm filtration and ultracentrifugation effectively eliminates live bacteria from the purified SsEVs fraction.
